# Social inequalities in the utilization of outpatient psychotherapy: analyses of registry data from German statutory health insurance

**DOI:** 10.1186/s12939-017-0644-5

**Published:** 2017-08-16

**Authors:** Jelena Epping, Denise Muschik, Siegfried Geyer

**Affiliations:** Hannover Medical School, Medical Sociology Unit, Carl-Neuberg-Str. 1, 30625 Hannover, Germany

**Keywords:** Social inequality, Outpatient psychotherapy, Use of health services

## Abstract

**Background:**

Most studies on health disparities deal with the occurrence of disease, but little is known about inequalities in the utilization of mental health services. This paper examines social inequalities in the utilization of outpatient psychotherapy within a health care system where there are low financial barriers to health care and a lack of specific health policies to address access to psychotherapeutic services.

**Methods:**

Registry data of German statutory health insurance for the year 2013 were used (total population: *N* = 746,963; 10,711 women and men with psychotherapy). Logistic regression analyses were performed to estimate the effects of three socio-economic (SES) indicators on the utilization of psychotherapy.

**Results:**

Utilization of psychotherapy by SES status did not correspond to the social structure of the insured population. Social disparities that disadvantaged less privileged women and men were found; this applied to education, income and occupational position. The most pronounced differences were found for education. In contrast, effects of income were rather small. These findings must be interpreted against the backdrop of the absence of financial barriers to outpatient psychotherapy in Germany.

**Conclusions:**

A marked degree of psychotherapy under-utilization was found for lower SES groups. Psychotherapists should pay increased attention to clients with lower socio-economic position. Enhancing mental health literacy, as well as reducing the stigma of mental illness, is crucial for increasing the usage of psychotherapeutic services of those who need it most. Relevant health policy is needed to reduce the barriers to, and consequently increase psychotherapy utilization.

## Background

The evidence of social inequalities in psychiatric illness [1–3] and physical diseases [4, 5] is well established. The highest health risks are consistently found for women and men with the lowest educational degrees, the lowest occupational positions, and the lowest incomes [4, 5]. These relationships are not to be understood as categorical, but as gradual; therefore, in the literature it is called the “social gradient.” Independent of social position health care should cover all social groups according to their needs. According to the principles of the welfare state (Art. 28 of the Basic Law of Germany), unequal treatment for ill or impaired persons, especially in terms of economic inequality, is not permitted in Germany [6]. Research shows, however, that different patterns of health services utilization exist among socio-economic groups for physical impairments [7, 8] and psychiatric illnesses [9–12]. For Germany, the research body on social inequality in mental health services utilization for persons with psychological problems is however scarce.

For the majority of psychological problems, psychotherapy is the method of choice, or at least an indispensable part of the treatment plan. Still, according to the Network for Psychotherapeutic Care in Europe, the utilization of psychotherapy is infrequent [13]. In seven out of 20 European countries (Czech Republic, Finland, Germany, Hungary, Italy, Poland and the UK), there is full coverage of costs for psychotherapy patients [14]. The remaining 13 countries provide only partial or no coverage at all, thus making utilization of psychotherapeutic services dependent on the financial resources of the patient. This paper analyzes the utilization of psychotherapy in Lower Saxony, Germany, in a health care setting without financial barriers. As for other types of medical treatment, the utilization of psychotherapy should not depend on socio-economic status of a person. There are however associations with knowledge about mental illness: according to the British Attitude Survey higher socio-economic position was associated with higher levels of knowledge about mental illness [15]. Rüsch and colleagues on the other hand found that help-seeking for a mental health problem was associated with high degrees of mental health literacy [16]. Thus, one may assume that socio-economic status has an influence on utilization of health services even in a system claiming equal access to care.

Knowledge about the utilization of outpatient psychotherapy is scarce, and usually studies are based on survey data. In a German study Albani et al. [17] interviewed 1212 respondents about their access to psychotherapy and about their treatment. The sample was drawn after a screening procedure that was embedded in a population survey. Respondents were asked whether they underwent treatment by a psychotherapist within the last 6 years before the interview. Respondents with higher amounts of education (>10 yrs) were more likely to have psychotherapy than the general population. However, no further comparisons by socio-economic categories were performed.

Another study used data from the British Household Panel Survey from the years 1991 to 2009 [18]. A sample of 28,054 individuals was included, and 2410 individuals reported undergoing psychotherapeutic treatment. Household income, education and occupational status were available for differentiating respondents by socio-economic position. Respondents of the highest income quintile were less likely (OR = 0.43) than those of the lowest income group to use psychotherapy provided by the National Health Service (NHS). In contrast, the likelihood of utilizing psychotherapy provided outside the NHS was much higher in subjects with the highest educational level (OR = 6.51) compared to the lowest education group. When interpreting these results, it must be kept in mind that approximately 78% of all psychotherapy treatments in the study were provided within the NHS-framework, but psychotherapy from the NHS is not as readily available as private treatment. Patients first need a referral from their general practitioner, and they must wait several months before being treated [18].

As a part of the Study of Health in Pomerania, Germany, a survey on depression was carried out from 2007 to 2010. Schomerus and colleagues examined three groups of predictors for help-seeking in subjects with major depressive disorders [19]. It was reported that women and men with higher education were more likely to ask for treatment than those with less than 10 years of schooling (odds ratios: 2.18-2.93). In contrast, income did not predict help-seeking behavior.

All of the studies reported above were based on survey data. In our own study, we used a different approach by examining the effect of social inequalities on the utilization of outpatient psychotherapy by using health insurance data. This sort of database has the advantage of large case numbers and completeness of records, and due to the nonreactive nature of the data, problems of selective non-response do not emerge.

In Germany, psychotherapeutic treatment is covered by statutory health insurance (SHI) since 1967. According to the current guidelines, statutory health insurance covers three types of psychotherapy: behavior therapy, psychoanalysis, and psychodynamic therapy. For members of statutory health insurance (approximately 90% of German residents), there are no financial barriers for psychotherapeutic treatment. For persons receiving private health insurance, access depends on the health care plan negotiated between companies and clients.

Psychotherapeutic treatment can be performed and covered by the SHI for the following diagnoses: mood [affective] disorders (ICD10-Codes: F32, F33, F34.1), neurotic disorders, stress-related and somatoform disorders (F40-F45), behavioral syndromes associated with physiological disturbances and physical factors (F50-F52), disorders of adult personality and behavior (F60-F69), and behavioral and emotional disorders with onset usually occurring in childhood and adolescence (F90-F98). After five preparatory sessions dedicated to evaluation before starting therapy, an application for SHI must be completed. In case of approval being received, which is the case for over 90% of instances, the psychotherapeutic treatment can start.

Taking higher prevalence rates of mental disorders in lower socio-economic groups into account, we expected utilization rates to be distributed accordingly. The following research questions were dealt with:

1. Are there social disparities in the utilization of outpatient psychotherapy?
2. Do all three indicators of socio-economic position (education, income, and occupational position) independently account for social disparities?

We assumed utilization rates of outpatient psychotherapy to be higher in lower socio-economic groups. Moreover, we expected education to have the strongest effects on utilization of psychotherapy, since education was shown to be the main explanatory factor of mental health literacy [15].

## Methods

The analyses are based on an anonymized dataset from 2013 provided by AOK Niedersachsen, a large SHI provider in the state of Lower Saxony in Germany. In Germany, health insurance is mandatory to all inhabitants, in 2015 only about 0,1% were uninsured [20]. Residents below a yearly pre-tax income of about 56,000€ are automatically enrolled into the statutory system. In the year 2015 about 88% of all residents were insured in a SHI [20].

The distributions of gender and age in the population from the AOK Niedersachsen dataset were similar to the population of Lower Saxony and of Germany as a whole [21]. However, the insurance population differed from those of Lower Saxony with respect to the occupational and educational structures. Therefore, the population of AOK Niedersachsen cannot be considered a representative sample for the population of Lower Saxony or Germany, and the data must be considered as representing a distinct population. The data were originally collected for accounting purposes; thus, the only treatments recorded were those covered within the system of statutory health insurance. The preparation of the dataset was subject to a data project, where it was thoroughly checked for errors, consistency, duplicates, and the correctness of the temporal order of events. With these procedures, the data were made suitable for scientific purposes. The work was done according to the guidelines in the “Good Practice of Secondary Data Analysis (GPS)”.

In addition to data on treatment, the insured individuals can be described by gender, age, and socio-economic position. Information on the latter was available for those who were employed and insured. According to regulations, employers must annually report qualification level, occupation, and individual income of their employees insured in a SHI to their health insurance provider. These data are then entered into the federal statistics.

As only employed, insured individuals could be classified by socio-economic position, analyses were restricted to this group. In addition, the age range was confined to individuals between 22 and 59 years. Below the age of 22 and above 59, a large proportion of insured individuals were not employed, thus information on socio-economic position was unavailable.

There are three indicators of socio-economic position available in the data: qualification, occupational position and income. In the analysis, all three available indicators were used, as they are only moderately correlated and have different latent contents [5, 22, 23]. Moreover, low-income individuals may experience several specific obstacles in access to psychotherapy. Krupnick and Melnikoff [24] have discussed practical barriers in low-income groups in terms of transportation problems, lack of child care, work scheduling conflicts, as well as cultural and psychological barriers such as speaking not the same language as the psychotherapist, mistrust to authorities and potential cultural differences due to differing social and cultural backgrounds between patient and medical personnel. Therefore income had to be included in spite of absence of direct financial barriers in access to psychotherapy.

The following indicators of socio-economic position were used for predicting utilization rates:

- Qualification was used as an indicator of socio-economic position in terms of vocational training. The available categories were “without vocational training,” “with vocational training” and “university degree.” We used this indicator instead of school education because it depicts the highest educational level obtained.
- Occupational groups were formed based on a classification system of occupations by Blossfeld [25]. The system contains 12 occupational classes comprising occupations that are homogenous with respect to school and vocational training as well as occupational activities. For the present analysis, 12 groups were combined into four: unskilled, skilled, specialists and highly qualified. “Specialists” differed from “skilled” employees by higher qualification and more advanced decision latitude. For falling into the “highly qualified” group, a person usually needed a university degree, and the occupation included management tasks.
- Income was available as individual pre-tax income. It had been recommended to use household income [26, 27], but in comparative analyses, individual income can be used [28].
- For our analysis, income was classified into three groups based on the average income in Germany using data from the Federal Statistical Office. The lowest income group comprised pre-tax annual income of <40% of the average income in Germany (<12′000 €), the highest income group was above 80% of the AI-G (more than 24′000 €), and the middle group was in between.

Analyses were performed separately for women and men, as well as for two age groups, 22–40 and 41–59 years. We expected differences in the influence of SES on utilization dependent on age. Due to multiple stratifications in the logistic regression model, no more than two age groups were created.

Data on frequency of usage of outpatient services were used to examine the utilization of psychotherapeutic treatment. The dependent variable was utilization of outpatient psychotherapy (at least one session). As described above, five preparatory sessions took place before starting regular therapy, followed by an application to the SHI. Only in the case of an agreement between the patient and psychotherapist, and only after approval from the SHI, outpatient psychotherapy started.

Statistical procedures were done using STATA 11.1MP. Significance tests for crosstables were performed with a Chi^2^-test by using a significance level of *p* < 0.05. Odds ratios and corresponding confidence intervals are reported. Standard errors for calculating confidence intervals were obtained by drawing 500 bootstrap samples, thus leading to normally distributed residuals.

## Results

The descriptives of the relevant variables are displayed in Table 1. Overall, 2.4% of the employed women and 0.7% of the employed men aged 22–59 had at least one psychotherapeutic treatment in 2013 after successful application. Distributions of psychotherapy patients over educational or occupational groups differed from the total insured population. The proportion of individuals with university degree, as well as specialists and persons in highly qualified occupations, was significantly higher among the psychotherapy clients.

**Table 1:** Descriptives of the insured individuals and psychotherapy patients, with employment status, aged 22–59, in the AOK-Niedersachsen 2013

		Insured			Psychotherapy patients		
		male	female	total	male	female	total
*N*		426,069	320,894	746,963	3066	7645	10,711
Age Chi^2^(df = 1) = 5.06, *p* = 0.02	22-40	47%	45%	46%	43%	46%	45%
	41-59	53%	55%	54%	57%	54%	55%
Qualification Chi^2^(df = 3) = 984.48, *p* < 0.001	no voc.training	15%	16%	15%	10%	11%	11%
	with voc.training	71%	66%	68%	74%	72%	73%
	university degree	3%	5%	4%	9%	9%	9%
	missing	11%	13%	12%	7%	8%	8%
Occupational group Chi^2^ (df = 3) = 1422.00, *p* < 0.001	unskilled	22%	28%	25%	15%	18%	17%
	skilled	58%	31%	46%	50%	33%	38%
	specialist	15%	35%	23%	23%	40%	35%
	highly qualified	5%	6%	6%	11%	10%	10%
	missing	0%	0%	0%	0%	0%	0%
Individual income Chi^2^ (df = 3) = 304.65, *p* < 0.001	low	6%	22%	13%	6%	17%	14%
	middle	25%	39%	31%	19%	32%	28%
	high	53%	21%	39%	51%	28%	35%
	missing	16%	19%	17%	24%	23%	23%
persons with at least one psychotherapeutic treatment	*n*	3066	7645	10,711			
	% on all insured	0.7%	2.4%	1.4%			

**Fig. 1:**
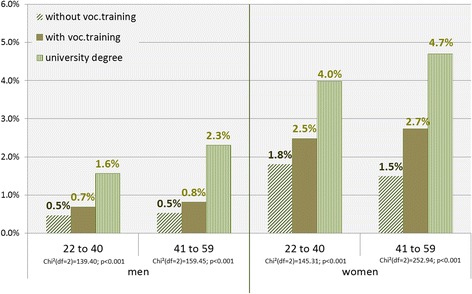
Utilization of psychotherapy by qualification level, gender, and age groups

Stratified by qualification level and by age groups, marked differences among the examined groups emerged. In insured individuals with the highest qualification (university degree), above-average rates of psychotherapeutic treatment (Fig. 1) were observed. This applied to both age groups and to men and women alike, though in insured women above 40, differences between the middle and the highest qualification group were more apparent.

**Fig. 2:**
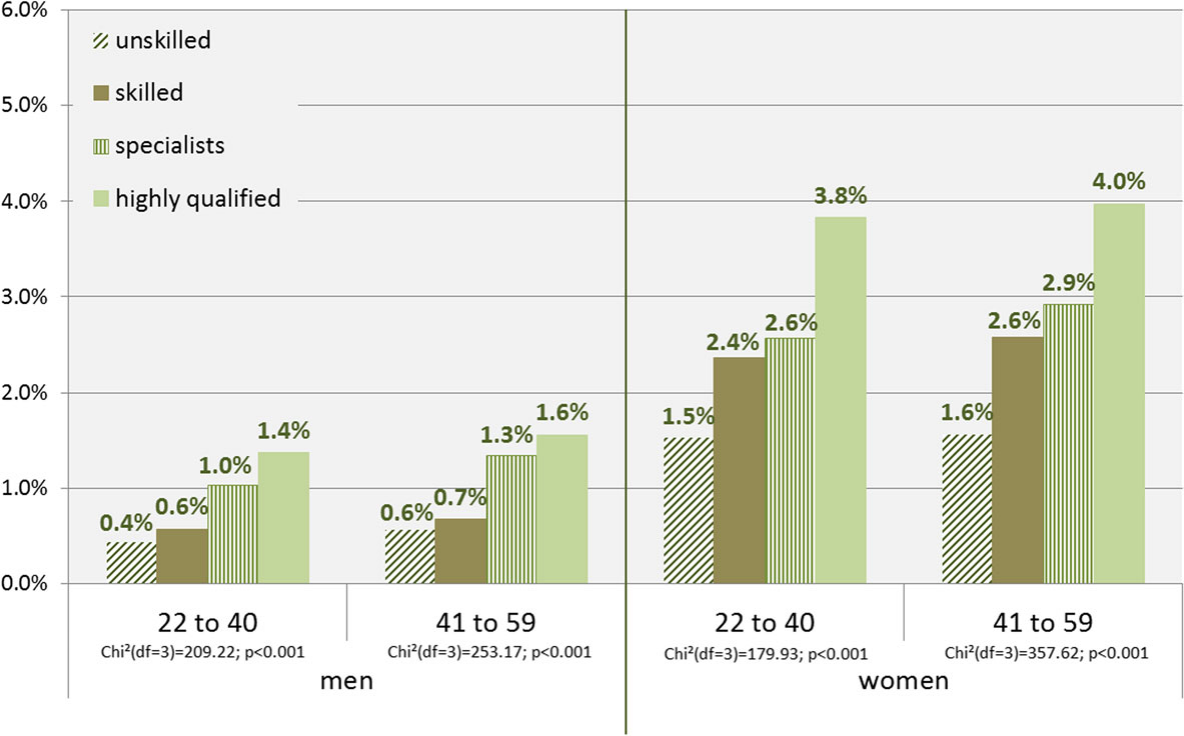
Utilization of psychotherapy by occupational qualification, gender, and age groups

Comparing the four occupational groups, similar findings were found only in women, as the elevated utilization rates were most pronounced in the highly qualified group (Fig. 2). In men, the largest difference emerged between the second and third occupational groups, between skilled employees and specialists. Still, there was a clear gradient in the utilization of psychotherapy towards the higher occupational group for both age categories, men and women alike.

**Fig. 3:**
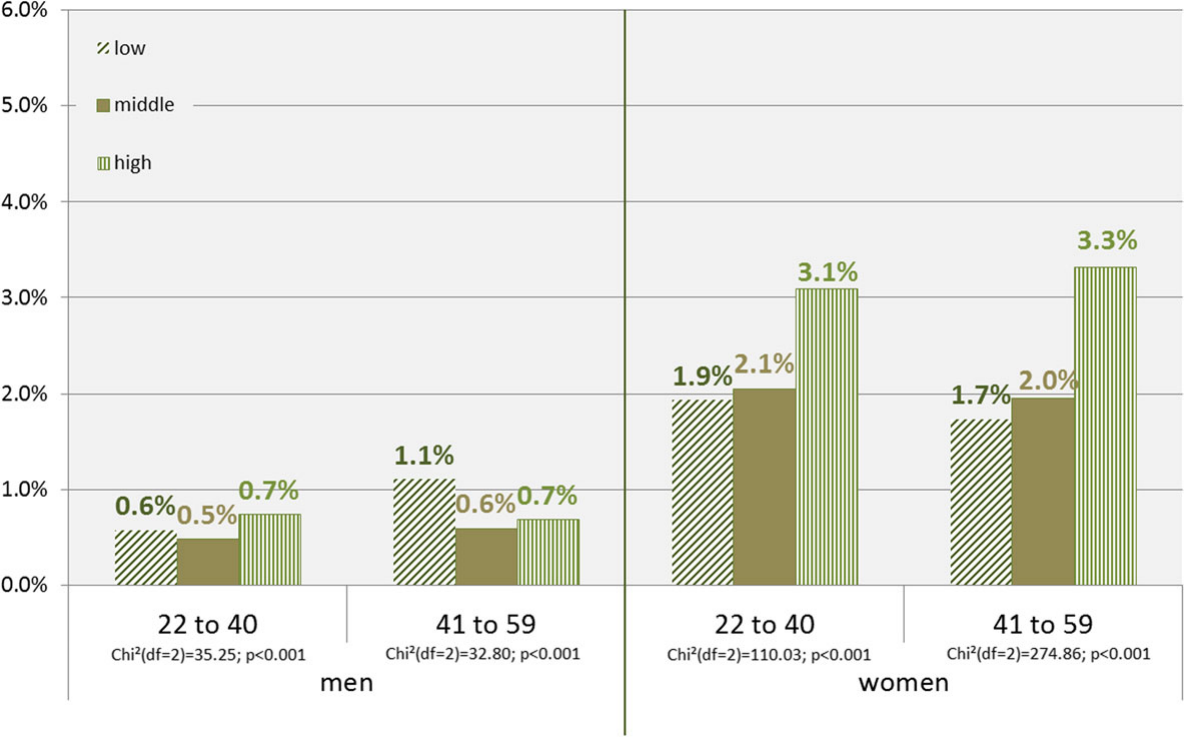
Utilization of psychotherapy by individual income, gender, and age groups

For individual pre-tax income, the gradient towards the higher income group emerged only in women. Utilization rates were almost twice as high in the highest income group aged 41–59 than in the lowest income group (Fig. 3). In men aged 41 to 59, inverse utilization rates for psychotherapy emerged, as the lowest income group had increased rates of psychotherapeutic treatment. For men aged 22–40 years, there was almost no difference in the utilization rates between income groups.

The descriptive results above displayed the utilization rates for each indicator, although the dimensions of discrepancies in the distribution of SES indicators between psychotherapy patients and the whole population remain unclear. The three indicators correlated weakly to moderately: Spearman’s rho was r_s_ = 0.10 between occupational group and income, r_s_ = 0.21 between income and qualification, and r_s_ = 0.30 between occupational group and qualification (*p* < 0.001 for all coefficients).

On the basis of these weak to moderate correlations, the regression model was computed. All SES-indicators were included to estimate their independent effects on psychotherapeutic treatment. Furthermore, the SES-indicator with the strongest effect could be determined. Separate analyses were performed for men and women as well as for the two age groups. Odds ratios are presented in Table 2.

**Table 2:** Logistic regression on utilization of psychotherapy by qualification, occupational position, and individual income, stratified for gender and age

	men					
	22-40 yrs			41-59 yrs		
	OR	95%-CI	*p*	OR	95%-CI	*p*
Qualification						
Ref: no vocational training	1	--	--	1	--	--
Vocation training	1.33	1.06 – 1.68	0.014	1.39	1.14 – 1.69	0.001
University degree	2.05	1.50 – 2.80	<0.001	2.87	2.12 – 3.87	<0.001
Occupational position						
Ref: unskilled	1	--	--	1	--	--
skilled	1.08	0.87 – 1.34	0.477	1.13	0.95 – 1.33	0.164
specialist	1.99	1.58 – 2.50	<0.001	2.08	1.71 – 2.53	<0.001
highly qualified	2.12	1.59 – 2.80	<0.001	2.12	1.67 – 2.70	<0.001
Pre-tax income						
Ref: high	1	--	--	1	--	--
middle	0.85	0.73 – 1.00	0.045	1.00	0.87.- 1.15	0.957
low	1.40	1.08 – 1.83	0.012	2.22	1.78 – 2.76	<0.001
	women					
	22-40 yrs			41-59 yrs		
	OR	95%-CI	*p*	OR	95%-CI	*p*
Qualification						
Ref: no vocational training	1	--	--	1	--	--
Vocation training	1.10	0.95 – 1.27	0.195	1.50	1.34 – 1.70	<0.001
University degree	1.48	1.22 – 1.80	<0.001	2.17	1.79 – 2.61	<0.001
Occupational position						
Ref: unskilled	1	--	--	1	--	--
skilled	1.52	1.29 – 1.79	<0.001	1.38	1.25 – 1.54	<0.001
specialist	1.57	1.33 – 1.84	<0.001	1.49	1.34 – 1.65	<0.001
highly qualified	1.78	1.44 – 2.19	<0.001	1.55	1.32 – 1.82	<0.001
Pre-tax income						
Ref: high	1	--	--	1	--	--
middle	0.77	0.70 – 0.85	<0.001	0.68	0.62 – 0.73	<0.001
low	0.78	0.70 – 0.88	<0.001	0.68	0.61 – 0.75	<0.001

For the male insured individuals, a marked social gradient emerged for both age groups, with an odds ratio of OR = 2.05 (22–40 yrs.) and 2.87 (41–59 yrs) for qualification. Similar findings were obtained for occupational position, but the odds ratio for the older group was smaller than for qualification. For income, no difference between the high and middle income emerged. The low-income group showed the highest level of risk in both age groups.

The findings in women were reproduced in men, and this applied to qualification and occupational position, although at lower levels. The odds ratios of income also indicated social differences, i.e., those with the highest incomes were most likely to undergo psychotherapy, but the lowest and the intermediate income groups did not differ in that respect. Thus, there were no clear social gradients for income.

## Discussion

In this study differential access to outpatient psychotherapy was analyzed in terms of socio-economic background of individuals within statutory health insurance. This included vulnerable groups as far as they were employed. In accordance to well-established finding of social inequalities in mental illness, we expected that the utilization of psychotherapy would follow the same pattern. Thus, the likelihood of assessing psychotherapeutic treatment might increase with decreasing income, education, and occupational position. However, the current findings indicated the opposite, as those with the highest risk of mental illness had the lowest utilization rates, thus leading to multiple and cumulative disadvantages. Our results confirm the earlier analyses of Albani et al. [17] and Schomerus et al. [19], and taken together, they indicate a marked under-coverage of psychotherapeutic treatment in lower socio-economic groups.

The second question referred to the relative strengths of the effects of the three indicators of social differentiation. Qualification was the strongest predictor of utilization, while the effects of occupational position were less strong, but in both cases social gradients emerged. A possible explanation for these findings may be differential verbalization skills that could be disadvantageous for building a therapeutic alliance between therapist and client, which might be correlated with educational status [24]. Mental health literacy [29] is a second possible explanation for these findings. Thornicroft states that the “lack of knowledge about features and treatability of mental illnesses” increases the likelihood of treatment avoidance [30]. In accordance with this assumption, Rüsch and colleagues found that help-seeking following the onset of mental health problems correlated with increased knowledge about mental illness [16]. The British Attitudes Survey [15] produced evidence that higher socio-economic position was associated with higher levels of mental health literacy and lower levels of stigma, especially in women.

In contrast to occupational position and qualification, effects of income were present, but less consistent with respect to a social gradient. As Krupnick and Melnikoff have stated [24], several barriers to care for low-income patients may occur in spite of overt financial barriers being absent. On the one hand, practical issues like transportation costs, lack of child care or work scheduling conflicts may keep patients from utilizing regular psychotherapeutic treatment. On the other hand, psychological and cultural barriers such as stigma of mental illness, mistrust of authority, differing cultural and social backgrounds between patient and mental health specialist might prevent them from utilizing psychotherapy.

### Limitations

The discussion of obstacles to psychotherapy points to a possible weakness of our dataset. Our data do not permit depiction of the social gradient of the whole population of Lower Saxony or Germany, as the highest 10% of wage earners and individuals employed by the state are privately insured. This leads to an underestimation of the social gradient of the whole insured population.

These disadvantages must be weighed against the advantages of our data, i.e., that all psychotherapies paid by the statutory health insurance are recorded, the data are not impaired by non-response, which is especially relevant for ill persons, and memory or response biases are absent.

## Conclusions

The study revealed a marked under-coverage of outpatient psychotherapeutic treatment in lower socio-economic groups. Qualification played an important role in predicting psychotherapy utilization rates. An important implication of our findings is that psychotherapists should pay increased attention to clients with lower socio-economic position, and therapies should be adapted to clients who are verbally less fluent. On a population level, mental health literacy should be improved to lower the barriers to, and consequently increase psychotherapy utilization. Broadening knowledge about features and treatability of mental illnesses, especially for persons with lower education, must become part of health policy.
